# Comparative analysis of geriatric hip fracture management outcomes in teaching and nonteaching hospitals in Jordan

**DOI:** 10.1038/s41598-024-66016-x

**Published:** 2024-07-11

**Authors:** Moh’d S. Dawod, Mohammad N. Alswerki, Anas AR Altamimi, Mohammad Abu Hilal, Ashraf Albadaineh, Yaser Saber, Mohammed S. Alisi, Jihad Al-Ajlouni

**Affiliations:** 1 Faculty of Medicine, Mutah University, Al-Karak, Jordan; 2 Department of Orthopedic Surgery, Jordan University Hospital, P.O. Box: 13046, Amman, 11942 Jordan; 3 Head of Special Surgery Department, Hashemite University, Amman, Jordan; 4 Al-Karak Governmental Hospital, Jordanian Ministry of Health, Amman, Jordan; 5 Jordanian Ministry of Health, Amman, Jordan; 6 Islamic University of Gaza, Palestinian Ministry of Health, Gaza, Palestine; 7 Jordan University Hospital, Amman, Jordan

**Keywords:** Geriatric hip fractures, Hip fractures, Teaching hospitals, Postoperative complications, Hip fracture outcomes, Hip fracture mortality, Trauma, Medical research, Musculoskeletal system, Disability, Risk factors, Health care, Fracture repair, Geriatrics, Health policy, Health services, Prognosis, Public health

## Abstract

Hip fractures are common orthopedic injuries that have significant impacts on patients and healthcare systems. Previous studies have shown varying outcomes for hip fracture management in different settings, with diverse postoperative outcomes and complications. While teaching hospital settings have been investigated, no studies have specifically examined hip fracture outcomes in teaching hospitals in Jordan or the broader Middle East region. Therefore, the aim of this study was to investigate this important outcome. A cohort comprising 1268 patients who underwent hip fracture fixation from 2017 to 2020 was analyzed for nine distinct outcomes. These outcomes encompassed time to surgery, ICU admissions, perioperative hemoglobin levels, length of hospital stay, readmission rates, revision procedures, and mortality rates at three time points: in-hospital, at 6-months, and at 1-year post-surgery. The analysis of 1268 patients (616 in teaching hospitals, 652 in non-teaching hospitals) showed shorter mean time to surgery in teaching hospitals (2.2 days vs. 3.6 days, *p* < 0.01), higher ICU admissions (17% vs. 2.6%, *p* < 0.01), and more postoperative blood transfusions (40.3% vs. 12.1%, *p* < 0.01). In-hospital mortality rates were similar between groups (2.4% vs. 2.1%, *p* = 0.72), as were rates at 6-months (3.1% vs. 3.5%, *p* = 0.65) and 1-year post-surgery (3.7% vs. 3.7%, *p* = 0.96). Geriatric hip fracture patients in teaching hospitals have shorter surgery times, more ICU admissions, and higher postoperative blood transfusion rates. However, there are no significant differences in readmission rates, hospital stays, or mortality rates at various intervals.

## Introduction

Hip fracture among the elderly population is considered a public health burden^1–4^. In the context of osteoporosis, geriatric populations are vulnerable to different fragility fractures, including vertebral compression fractures, distal radius fractures, and proximal humerus fractures^5^. Geriatric hip fractures pose a treatment challenge for both orthopedic surgeons and the health care system^6,7^. For orthopedic surgeons, it’s of paramount importance to obtain a rigid, stable fixation to allow early weight bearing, which is critical for enhanced recovery postoperatively^8^. Health care challenges encountered in these patients include the presence of multiple medical comorbidities, polypharmacy, and decreased physiological reserve, all of which can have a profound impact on health care system utilization and patients’ outcomes^9,10^.

In geriatric hip fractures, the treatment goals usually focus on enhanced recovery through proper preoperative patient optimization and stabilization of medical comorbidities, prevention of any unnecessary surgical delay, and a multidisciplinary approach for optimal outcomes^11–13^. Despite the maximal efforts involved in the care of these patients, the morbidity and mortality figures are relatively high^14^. Complications can range from surgery-related factors like infections and metal failure to medical issues such as venous thromboembolic events, cardiopulmonary complications, and even mortality^15^. The estimated one-year mortality in these patients can reach up to 36% even with surgical fixation^16^.

Previous studies have shown that the outcome of geriatric hip fractures can depend on the health care setting where management is taking place^6,17,18^. Factors such as the availability of specialized equipment^19^, adherence to standardized protocols^20^, and the composition of healthcare teams^21^ may differ between teaching and non-teaching hospitals. Therefore, comparing outcomes between these settings is essential to gain insights into the differences in care delivery. Teaching hospitals, often associated with medical education and research, may offer a wider array of specialized services and resources due to their role as academic centers^22^. Conversely, non-teaching hospitals may prioritize efficiency and cost-effectiveness in their care delivery mode^23^. By examining outcomes in both types of hospitals, researchers can better understand the impact of these different approaches on patient outcomes, leading to improvements in healthcare quality and informed policy decisions.

To the best of the author’s knowledge of currently available literature, no previous studies have discussed the outcome of geriatric hip fractures in teaching versus non-teaching hospitals in Jordan or the broader Middle East region, despite the health burden and the critical importance of this topic. Therefore, the aim of this study is to examine the treatment outcomes of geriatric hip fractures in teaching versus non-teaching hospitals in Jordan. Investigating this important topic will provide empirical evidence regarding the current status of geriatric hip fractures in teaching versus non-teaching hospitals in Jordan and will guide future research across this vital topic across the Middle East.

## Patients and methods

### Study design

This research employed a retrospective cohort and comparative design to examine specific outcomes in geriatric patients who underwent hip fracture fixation in teaching and non-teaching hospitals in Jordan from January 2017 to December 2020.

### Study setting

The study was conducted across two university-based teaching hospitals and two non-university-based governmental hospitals in Jordan. Patients were not specifically assigned to any hospitals; rather, they sought treatment at these hospitals based on their own preferences, which were influenced by factors such as geographical location, insurance coverage, and healthcare costs and accessibility.

### Study participants

A total of 1268 patients were included in this study. The inclusion criteria encompassed patients with geriatric hip fractures (intertrochanteric fractures and basocervical femur neck fractures) and underwent surgical fixation within the predetermined study period (January 2017–December 2020), and patients had to have sustained the fracture due to a low-energy fall. Conversely, patients meeting the exclusion criteria were those who experienced high-energy trauma, were treated nonoperatively, or had fractures other than those specified in the study.

### Outcome variables definitions

The primary objective of this study was to investigate the differences in selected outcomes between these two types of healthcare facilities. A total of nine outcome measures were utilized to facilitate the comparison between the two groups. These outcomes can be categorized into primary outcomes, reflecting patient-related measures, and secondary outcomes, which gauge the efficiency and performance of the healthcare system.

*Time to surgery* the number of days required from hospital admission to the day of surgery.

*Postoperative ICU admission* the occurrence of intraoperative or postoperative complications necessitating admission to the intensive care unit for stabilization.

*Pre and postoperative hemoglobin levels* measurement of patients’ hemoglobin levels both preoperatively (immediately upon hospital admission) and the drop in hemoglobin on postoperative day 1.

*Postoperative blood transfusion* defined as a significant drop in hemoglobin necessitating postoperative blood transfusion, with a cutoff point of a hemoglobin level of 8 mg/dl or a drop from the preoperative baseline accompanied by hemodynamic compromise.

*Length of hospital stay* the duration spent in the hospital for the management of geriatric hip fracture from admission to discharge to home, measured in days.

*VTE events* venous thromboembolic events (pulmonary embolism or deep venous thrombosis) causing symptoms and confirmed diagnosis with appropriate imaging modalities, necessitating administration of anticoagulant medications.

*Readmission within one month* hospital admission within one month of discharge regardless of the cause (either surgery-related or non-surgery-related).

*Need for revision* defined as any significant implant failure or implant-related complications causing significant symptoms and disabilities, necessitating revision surgery.

*Mortality* measured either in-hospital, within 6 months of surgery, or within 1 year of surgery performed for hip fracture.

### Data collection and sources

Data collection was conducted by obtaining authorized access to patients’ electronic health records and conducting patient interviews via phone calls. All patient data were securely stored and handled with privacy safeguards in place to protect patients’ confidentiality and privacy. Measures were taken to ensure that all identifiable patient information remained confidential throughout the data collection process.

### Bias control

In our study, several efforts were made to address potential sources of bias. Firstly, randomization was not feasible due to the retrospective nature of the study; however, efforts were made to minimize selection bias by including a diverse and representative sample of patients from different across in Jordan. Secondly, to mitigate information bias, standardized data collection methods were employed, and all data were collected from electronic health records and patient interviews using predefined way. Moreover, efforts were made to ensure consistency in data abstraction and interpretation by training research personnel involved in data collection.

### Sampling and study size

In this study, convenience sampling was utilized to gather data. The decision to include a sample size of 1268 patients was based on careful consideration of various factors. Firstly, previous studies with similar sample sizes were considered, ensuring consistency with established research practices. Additionally, technical factors, including the capacity and resources available at the participating hospitals, were considered in determining the appropriate sample size. This comprehensive approach aimed to ensure robust data collection while aligning with both methodological standards and practical constraints.

### Ethical considerations

An appropriate Institutional Review Board (IRB) approval was obtained for this study from the Jordan Ministry of Health General Ethics Committee for Scientific Research (IRB no. MOH/REC/2022/383). The Code of Ethics of the World Medical Association (Declaration of Helsinki) was followed while conducting the study. Informed consent was obtained from all patients prior to their participation in the study, adhering to ethical principles of autonomy and respect for individuals’ rights.

### Quantitative variables

Quantitative variables in this study were analyzed by comparing outcomes between patients treated in teaching and non-teaching hospitals. Groupings were based on hospital type. Variables such as mean age, smoking status, prevalence of comorbidities, waiting times for surgery, and postoperative hemoglobin levels were examined. Additionally, postoperative outcomes including ICU admission rates, blood transfusions, revision surgery, and mortality were compared.

### Statistical analysis

Statistical tests were utilized to evaluate the significance of observed disparities, elucidating the impact of hospital type on geriatric hip fracture management. Data were meticulously recorded and analyzed using statistical package for social science (SPSS), version 23. The analysis encompassed descriptive statistics and percentages, alongside the application of the student t-test to compare means between the two study groups. Additionally, bar charts were employed for visual representation. Statistical significance was determined using the *p* value, with values less than 0.05 considered indicative of statistical significance.

**Figure 1:**
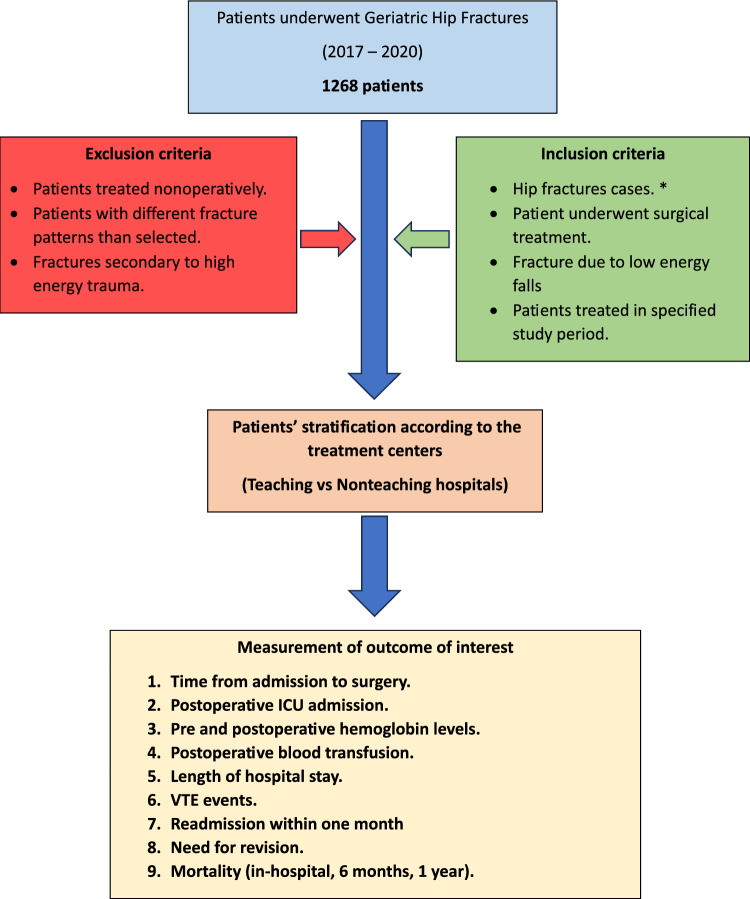
Flow chart of the study methodology. *Note fractures included intertrochanteric and basocervical fractures.

The study methodology is illustrated in the flowchart in Fig. 1.

### Ethical approval

This study received IRB approval (IRB no. MOH/REC/2022/383) from the Jordan Ministry of Health General Ethics Committee for Scientific Research, adhering to the World Medical Association’s Code of Ethics (Declaration of Helsinki).

### Informed consent

Informed consent was obtained from all subjects and/or their legal guardian(s).

## Results

The analysis encompassed a cohort of 1268 patients diagnosed with geriatric hip fractures, comprising 616 individuals treated within teaching hospitals and 652 within non-teaching hospitals. Within these cohorts, the mean age of patients treated in teaching hospitals was 76 years, while those in non-teaching hospitals averaged 74 years. Notably, a higher proportion of non-smokers was observed among patients in teaching hospitals (76.3%) compared to those in non-teaching hospitals (67.6%).

A comprehensive comparative overview of comorbidities, health profiles, gender distribution, and anesthesia type across both study groups is presented in Table 1.

**Table 1:** Health profiles among patients across the two study groups.

Comparison		Teaching hospitals (n = 616)	Non-teaching hospitals (n = 652)	*p* value
Health profiles		n. (%)	n. (%)	
Gender	Male	260 (42.2%)	327 (50.2%)	0.01*
	Female	355 (57.6%)	325 (49.8%)	
Smoking status	Smoker	146 (23.7%)	211 (32.4%)	0.001*
	Non-smoker	470 (76.3%)	441 (67.6%)	
Diabetes	Yes	312 (50.6%)	315 (48.3%)	0.40
	No	304 (49.4%)	337 (51.7%)	
HTN	Yes	424 (68.8%)	409 (62.7%)	0.02*
	No	192 (31.2%)	243 (37.3%)	
Cardiovascular diseases	Yes	218 (35.4%)	157 (24.1%)	< 0.01*
	No	398 (64.6%)	495 (75.9%)	
Cerebrovascular disease	Yes	124 (20.1%)	104 (16.0%)	0.05
	No	492 (79.9%)	548 (84.0%)	
Thyroid diseases	Yes	35 (5.7%)	20 (3.1%)	0.02*
	No	581 (94.3%)	632 (96.9%)	
Renal impairment	Yes	65 (10.6%)	39 (6.0%)	0.003*
	No	551 (89.4%)	613 (94.0%)	
Neurocognitive diseases	Yes	11 (1.8%)	29 (4.4%)	0.02*
	No	605 (98.2%)	623 (95.6%)	
Preop ICU need	Yes	24 (3.9%)	7 (1.1%)	0.001*
	No	592 (96.1%)	645 (98.9%)	
Anesthesia type	Spinal	412 (66.9%)	352 (54.0%)	< 0.01*
	GA	204 (33.1%)	300 (46.0%)	

Significant values are in bold.

**Figure 2:**
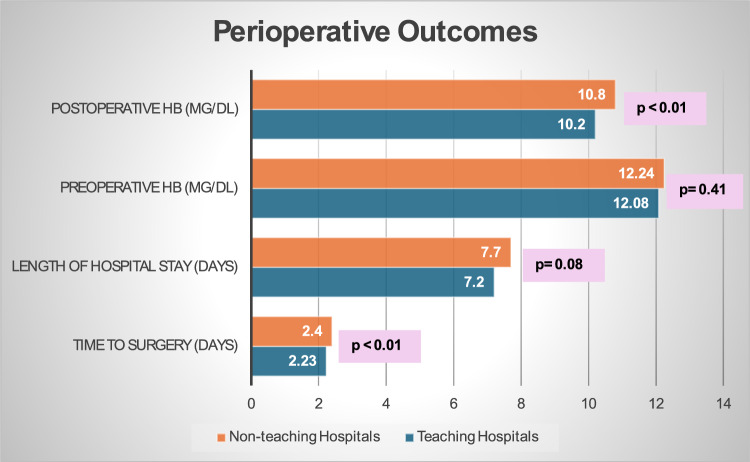
Bar chart illustration of perioperative variables.

The mean waiting times for surgery were 2.2 days in teaching hospitals and 3.6 days in non-teaching hospitals, yielding a significant *p* value of < 0.01, indicating a statistically significant difference. Similarly, postoperative hemoglobin levels differed between teaching (10.1) and non-teaching hospitals (10.7), with a *p* value of < 0.01. However, there were no statistically discernible differences in preoperative hemoglobin levels (*p* = 0.41) or lengths of hospital stays (*p* = 0.08). For a detailed overview, refer to Table 2, and Fig. 2 illustrates perioperative variables.

**Table 2:** Student t-test comparison between means of four outcome variables.

Comparison outcomes (means)	Teaching hospitals (n = 616)	Non-teaching hospitals (n = 652)	95% CI		*p* value
			Lower	Upper	
Time to surgery (days)	2.23	3.65	− 1.70	− 1.14	< 0.01*
Preop. hemoglobin (mg/dL)	12.08	12.24	− 0.54	0.22	0.41
Postop. hemoglobin (mg/dL)	10.15	10.78	− 0.81	− 0.44	< 0.01*
Length of hospital stay (days)	7.20	7.69	− 1.04	0.07	0.08

Significant values are in bold.

A comparative analysis of postoperative outcomes between the two study groups revealed significant differences. In teaching hospitals, 17% of patients required ICU admission due to medical deterioration, compared to 2.6% in non-teaching hospitals (*p* < 0.01). Similarly, postoperative blood transfusions were needed by 40.3% of patients in teaching hospitals and 12.1% in non-teaching hospitals (*p* < 0.01). Moreover, revision surgery rates were higher in teaching hospitals (4.4%) compared to non-teaching hospitals (1.7%) (*p* = 0.007). Detailed statistics can be found in Table 3.

**Table 3:** Chi-square test comparison of outcome measures between the two study groups.

Comparison of outcomes		Teaching hospitals (n = 616)	Non-teaching hospitals (n = 652)	Chi-square	*p* value
		n. (%)	n. (%)		
Postop. ICU admission		104 (16.9%)	17 (2.6%)	71.62	< 0.01*
VTE events		18 (2.9%)	11 (1.7%)	1.90	0.16
Need for blood transfusion		248 (40.3%)	79 (12.1%)	19.57	< 0.01*
One-month readmission		65 (10.6%)	68 (10.4%)	0.01	0.89
Need for revision		27 (4.4%)	11 (1.7%)	7.35	0.007*
Mortality	In-hospital	15 (2.4%)	14 (2.1%)	0.11	0.72
	6-months	19 (3.1%)	23 (3.5%)	0.19	0.65
	1-year	23 (3.7%)	24(3.7%)	0.002	0.96

Significant values are in bold.

The analysis of postoperative venous thromboembolic events, including deep vein thrombosis (DVT) and pulmonary embolism (PE), across the two study groups revealed a total of 18 events in the teaching hospitals group and 11 events in the non-teaching hospitals group (2.9% vs. 1.7% respectively, *p* value = 0.16). Similarly, the occurrence of readmission within one month from surgery, due to medical complications and health deterioration, was observed in 65 patients in the teaching hospitals group and 68 patients in the non-teaching hospitals group (10.6% vs. 10.4% respectively, *p* value = 0.89). These findings are summarized in Table 3.

**Figure 3:**
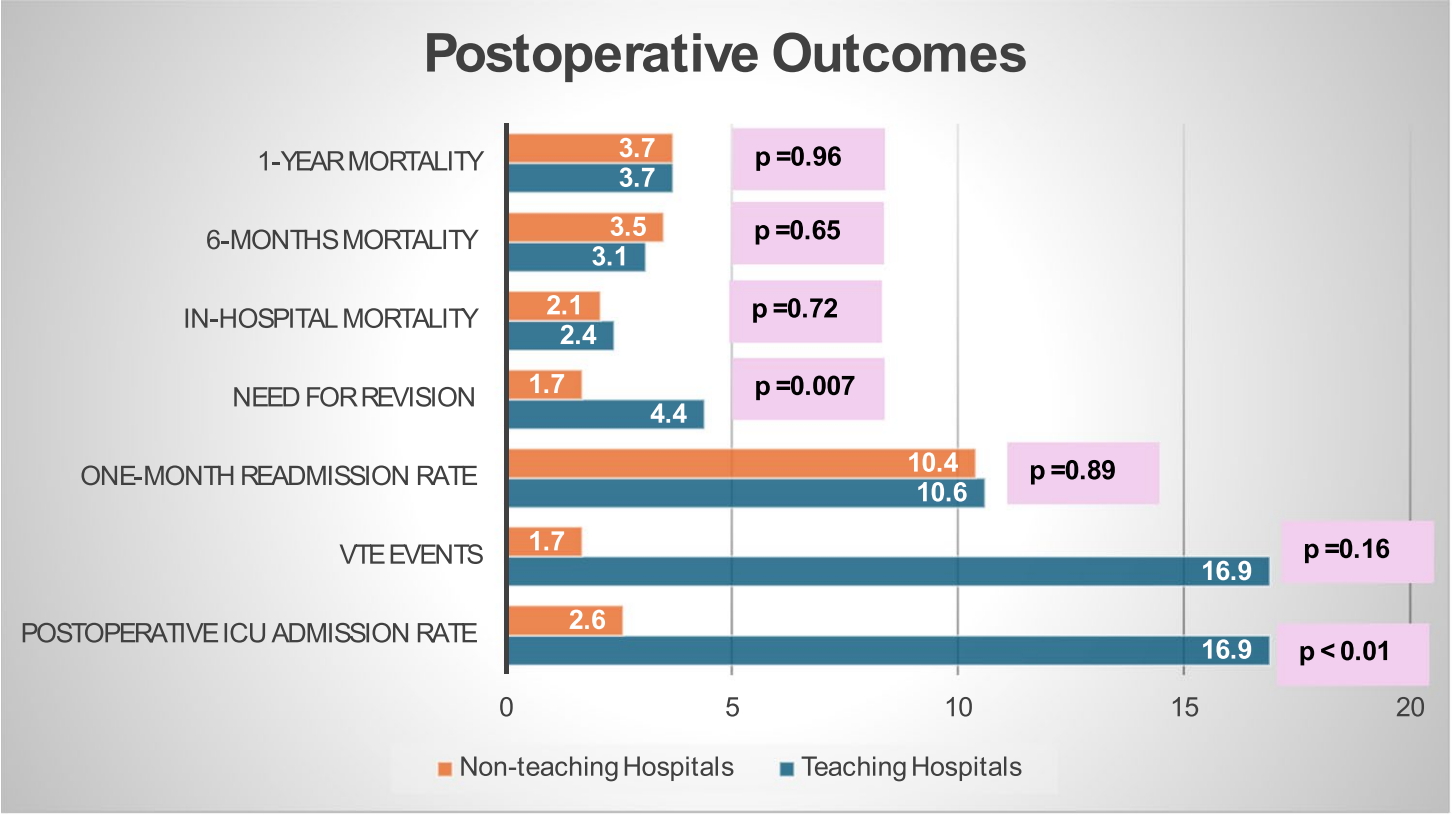
Bar chart illustration of postoperative outcomes.

In-hospital mortality rates were similar between the two groups: 15 deaths in teaching hospitals and 14 in non-teaching hospitals (2.4% vs. 2.1%, *p* = 0.72). Similarly, within 6 months post-surgery, there were 19 deaths in teaching hospitals and 23 in non-teaching hospitals (3.1% vs. 3.5%, *p* = 0.65). One-year mortality rates also showed no significant differences: 23 deaths in teaching hospitals and 24 in non-teaching hospitals (3.7% vs. 3.7%, *p* = 0.96). These findings suggest consistent mortality outcomes across specified time intervals. These findings are summarized in Table 3, and Fig. 3 is a bar chart illustration of selected postoperative outcomes.

## Discussion

The primary objective of this study was to examine potential disparities in the management of hip fractures between teaching hospitals and non-teaching hospitals. Additionally, the study aimed to assess the mediating effect of the teaching hospital setting in comparison to public non-teaching hospitals. By investigating these factors, the study sought to gain insight into the impact of hospital teaching status on hip fracture management and explore any potential differences that may exist.

Regarding the outcomes of interest identified in the study, patients receiving treatment in teaching hospitals demonstrated shorter time intervals to surgery, higher rates of postoperative ICU admissions, increased requirements for blood transfusions, and elevated revision rates. However, perioperative hemoglobin levels, occurrences of venous thromboembolic events (VTE), readmission rates, and mortality did not exhibit significant differences compared to patients treated in non-teaching hospitals.

When comparing our study findings to international standards such as those established by the National Hip Fracture Database and the National Falls and Fragility Fracture Adult Programme (FFFAP) 2023^24^, notable some emerge.

Firstly, the mean length of hospital stays in our study groups averaged around 7 days, considerably shorter than the approximately 16 days reported in the FFFAP data. This disparity can be attributed in part to the provision of postoperative rehabilitation in the FFFAP groups prior to discharge, contrasting with the more limited empirical rehabilitation provided in our study due to the absence of dedicated rehabilitation facilities. Secondly, analysis of anesthesia types revealed differences between our study groups and the FFFAP data. In teaching hospitals, 66.9% of patients received spinal anesthesia, compared to 54.0% in non-teaching hospitals and 53.5% in the FFFAP data. Conversely, the use of general anesthesia was more prevalent in non-teaching hospitals (46.0%) compared to teaching hospitals (33.1%) and the FFFAP data (48.1%). Finally, regarding revision rates, our study observed a higher revision rate in teaching hospitals (4.4%) compared to non-teaching hospitals (1.7%) and the FFFAP data (1.1%). This disparity may be attributed to surgeries performed by residents still in training in teaching hospitals, potentially leading to a higher reoperation rate. However, further research is warranted to delve deeper into this issue and explore potential contributing factors.

Hip fracture patients often exhibit frailty and multiple medical comorbidities, as evidenced by previous research. Boddaert et al. observed that 95% of elderly hip fracture patients present with at least one major comorbidity^25^. Härstedt et al. identified common postoperative comorbidities including hypertension, cognitive disorders, and ischemic heart disease^26^. Henderson et al. reported hypertension, osteoporosis, and ischemic heart disease as prevalent comorbidities in geriatric hip fracture patients^27^. In our study, encompassing both teaching and non-teaching hospital groups, hypertension and diabetes were the most prevalent comorbidities, while neurocognitive disorders were less common.

The literature concerning outcomes of hip fracture management in teaching hospitals is sparse, with limited evaluation of these outcomes. However, McGuire et al. found that patients treated in teaching hospitals exhibited a 1.4% decrease in 6-month mortality compared to those in non-teaching hospitals^28^. In our study, we observed a 0.4% lower mortality rate at 6 months in teaching hospitals compared to non-teaching hospitals. Similarly, Konda et al. reported associations between hip fracture management in teaching hospitals, shorter lengths of stay, and reduced in-hospital mortality^29^, findings that align with our study results.

In our study, the 1-year mortality rate was 3.7% for both teaching and non-teaching hospitals. This rate is significantly lower than the rates reported in previous studies on geriatric hip fracture management. Several observations can explain this discrepancy.

Firstly, the average age of patients in our cohort was 76 years for the teaching hospital group and 74 years for the non-teaching hospital group. These averages are lower than those reported in previous studies. For instance, Moyet et al. found the mean age for elderly patients with hip fractures to be 82.6 ± 7.4 years in a systematic review of the orthogeriatric care model^30^. Similarly, Basques et al. reported a mean age of 83.8 years in a study of 8434 patients^31^. Other studies have also reported higher mean ages, including Chen et al. (80.8 years)^32^, Krishnan et al. (81 years) ^33^, and Gleich et al. (85 years)^34^.

Secondly, a significant proportion of our patient cohort were non-smokers: three-quarters in the teaching hospital group and two-thirds in the non-teaching hospital group. Additionally, medical conditions linked to increased mortality, such as cardiovascular disease^35^, cerebrovascular disease^36^, renal impairment^37^, and neurocognitive diseases including dementia^38^, were less common in our cohort, affecting at most one-third of the patients.

Thirdly, we assessed the mortality rate using comprehensive data from both the Ministry of Health’s electronic health records and those of the teaching hospitals. This method allowed for accurate detection of mortality dates relative to the date of surgery. However, this number may still be underestimated. Some patients might seek care at hospitals outside these two major health sectors due to financial reasons or health insurance coverage issues, a phenomenon reported by Tewari et al. ^39^. Consequently, our study’s one-year mortality rate might not capture all deaths.

Therefore, combining the lower mean ages of our cohort, the lower prevalence of serious medical comorbidities, and the smaller number of smokers, along with considering that some patients may seek care outside the Ministry of Health and Teaching hospital health networks at the time of death, all contribute to the lower one-year mortality rates observed. However, a future follow-up study is in our scope to track mortality rates over longer time periods.

In our study, we observed that patients treated in teaching hospitals experienced significantly shorter times from hospital admission to surgery compared to those in non-teaching hospitals. Specifically, the time from admission to surgery was 2.2 days in teaching hospitals, while it was 3.65 days in non-teaching hospitals. This difference underscores the potential benefits of receiving care in teaching hospitals, where there may be greater resources and capabilities to promptly stabilize and optimize patients’ medical conditions prior to surgery. This efficient preoperative management likely contributes to the shorter time to surgery observed in teaching hospital settings.

In our study, hip fracture patients treated in teaching hospital settings were more likely to receive postoperative blood transfusions. This higher rate of transfusions in teaching hospitals suggests a lower threshold for administering blood products, possibly indicating a tendency towards more aggressive postoperative care practices.

Furthermore, an investigation and comparison of postoperative ICU admissions were conducted. Notably, there were no strict policy standards across the participating hospitals, with the decision to admit patients to the ICU depending upon various factors, including clinical status deterioration, laboratory and imaging assessments, evaluation by the ICU and medical teams, as well as consultations with relevant consultants. The observed higher rate of postoperative ICU admissions in teaching hospitals, while potentially associated with increased complication rates, also suggests a propensity for ICU utilization, indicating a heightened level of vigilance and attentiveness to patient needs within these settings. Collectively, these findings underscore the importance of comprehensive care and heightened vigilance characteristic of teaching hospitals. Contrary to concerns about increased hospital costs associated with heightened patient care in teaching hospitals, Konda et al. found no such association in the context of hip fractures^29^.

Previous research has examined the complications associated with hip fracture management in teaching hospitals, yielding conflicting results. Weller et al. reported a decreased risk of in-hospital mortality for hip fracture patients in teaching hospitals^40^. In contrast, Koval et al. reported higher in-hospital mortality rates and increased overall complications in hospital settings^41^. Similarly, Anderson et al. found a 3.6% increase in mortality among hip fracture patients in teaching hospitals compared to non-teaching hospitals^42^. Contrasting these findings, our study revealed that hip fracture patients treated in teaching hospitals experienced slightly higher rates of venous thromboembolic (VTE) events, comparable readmission rates, and similar mortality rates. These findings contribute to the existing literature by providing additional insights into the outcomes of hip fracture management in teaching hospitals, highlighting the need for further investigation and potential variability across different healthcare settings.

One limitation of this study pertains to its retrospective design and the relatively modest sample size of patients included. Despite these limitations, the study was able to draw meaningful conclusions and identify significant differences in findings. Another limitation is the absence of cost analysis, which was challenging to incorporate due to the complexity of healthcare insurance systems and variations in coverage within Jordan. However, it is worth noting that to the best of the authors’ knowledge, this study represents the first investigation into the management of hip fractures in teaching hospitals in Jordan and the Middle East.

## Conclusion

Geriatric hip fracture patients treated in teaching hospitals exhibit shorter times to surgery, heightened rates of postoperative ICU admissions, increased incidence of postoperative blood transfusions, and a greater likelihood of requiring revisions. Conversely, no significant differences were observed in terms of readmission rates, length of hospital stays, one-month readmission rates, or in-hospital, 6-month, and one-year mortality rates.

## Data availability

The datasets used and/or analysed during the current study available from the corresponding author on reasonable request.
